# Associations between air pollutant and pneumonia and asthma requiring hospitalization among children aged under 5 years in Ningbo, 2015–2017

**DOI:** 10.3389/fpubh.2022.1017105

**Published:** 2023-01-25

**Authors:** Xingyuan Zhou, Min Guo, Zhifei Li, Xiping Yu, Gang Huang, Zhen Li, Xiaohong Zhang, Liya Liu

**Affiliations:** ^1^Department of Pediatrics, The Affiliated Hospital of Medical School of Ningbo University, Ningbo, Zhejiang, China; ^2^Department of Obstetrics, Tangshan Maternal and Child Health Care Hospital of Hebei Province, Tangshan, Hebei, China; ^3^Department of Preventative Medicine, Medicine School of Ningbo University, Ningbo, Zhejiang, China

**Keywords:** air pollution, pneumonia, asthma, children, generalized additive model, time-series analysis

## Abstract

**Introduction:**

Exposure to ambient air pollutants is associated with an increased incidence of respiratory diseases such as pneumonia and asthma, especially in younger children. We investigated the relationship between rates of hospitalization of children aged under 5 years for pneumonia and asthma and the concentration of air pollutants in Ningbo between January 1, 2015 and August 29, 2017.

**Methods:**

Data were obtained from the Ningbo Air Quality Data Real-time Publishing System and the big data platform of the Ningbo Health Information Center. A generalized additive model was established via logarithmic link function and utilized to evaluate the effect of pollutant concentration on lag dimension and perform sensitivity analysis.

**Results:**

A total of 10,301 cases of pneumonia and 115 cases of asthma were identified over the course of this study. Results revealed that PM2.5, PM10, SO2 and NO2 were significantly associated with hospitalization for pneumonia and asthma in children under 5 years of age. For every 10-unit increase in lag03 air pollutant concentration, hospitalization for pneumonia and asthma due to PM2.5, PM10, SO2 and NO2 increased by 2.22% (95%CI: 0.64%, 3.82%), 1.94% (95%CI: 0.85%, 3.04%), 11.21% (95%CI: 4.70%, 18.10%) and 5.42% (95%CI: 3.07%, 7.82%), respectively.

**Discussion:**

Adverse effects of air pollutants were found to be more severe in children aged 1 to 5 years and adverse effects due to PM2.5, PM10 and SO2 were found to be more severe in girls. Our findings underscore the need for implementation of effective public health measures to urgently improve air quality and reduce pediatric hospitalizations due to respiratory illness.

## 1. Introduction

Pneumonia, the single largest infectious cause of death in children worldwide, accounts for 14% of all deaths of children under 5 years of age and killed 740,180 children in 2019 alone (1, 2). In China, the annual incidence of clinically severe pneumonia among this pediatric population decreased by 69% from 1990 to 2015 (3). However, despite this generally optimistic downward trend, mortality of pediatric pneumonia patents in China remains high. In 2015 alone, 22,242 children aged under 5 years died of pneumonia (4). Asthma incidence was also reported to have decreased worldwide (5). Despite declining asthma mortality, the global health burden of this condition remains significant. In 2015, ~334 million people worldwide were reported to be living with asthma, among them many school-age children (6, 7). The combined burden of pediatric pneumonia and asthma warrants scrutiny to achieve the 2030 Sustainable Development Goals (7, 8).

Over the past 20 years, numerous studies have shown that exposure to air pollution correlates with increased rates of respiratory and cardiovascular diseases, as well as mortality (9–11). Ambient particulate matter (PM) in air pollutants not only cause lung infections but also enter the circulation *via* the blood gas barrier, thereby inflicting widespread damage (12–16). Corrosive gaseous pollutants such as SO_2_ damage the skin, mucous membranes, and respiratory system (17). Exposure to air pollutants triggers and/or exacerbates lung disease in people of all ages (18, 19). Among them, children are particularly vulnerable to the adverse effects of air pollution and are more likely than adults to suffer from respiratory diseases related to air pollutants (20, 21). The reasons may be that children's ventilation rate is higher than that of adults, children have biological characteristics such as immature pulmonary and immune systems, and early exposure to air pollution may affect the development of children's respiratory and immune systems (22–24). As such, adverse health effects due to air pollutants among children aged under 5 years is particularly concerning. Although a number of studies evaluating effects of air pollution on pediatric pulmonary health have been performed worldwide (25–29), findings remain inconclusive. Importantly, levels of air pollution as well as socioeconomic development vary greatly from region to region. To date, few studies have evaluated associations between air pollution and pneumonia and asthma in children under 5 years old in China. Indeed, most similar research conducted in China has evaluated associations between only one type of air pollutant and pathology, with most studies having analyzed regions with remarkably high air pollution levels. As such, associations among several major air pollutants with pediatric respiratory pathology in regions with lower levels of air pollution warrant study.

Ningbo is a coastal city in China geographically located in the region of the Ningbo Plain and is characterized by relatively warm and humid year-round weather as well as a subtropical monsoon climate. Ningbo is also characterized by well-developed secondary industry and a lack of central heating. Between 2015 and 2017, excellent and good air quality rates were reported to have been 82.7, 84.7, and 85.2%, respectively. Over this same time period, the Chinese national comprehensive index of air quality ranked Ningbo 24th, 19th, and 17th among key cities and 6th, 5th, and 4th among the 25 most important Yangtze River Delta cities. Although the Ningbo ambient air quality remained stable and tended to be good, general pollution trends remained concerning as levels of particulate matter (PM) <2.5 micrometers in diameter (PM_2.5_) exceeded national secondary safety standards and levels of fine PM were high (30–32). Haze was also found to have increased levels of air pollution in colder months. In this study, Ningbo was selected to evaluate associations between concentrations of six ambient pollutants (PM_2.5_, PM_10_, O_3_, CO, SO_2_, and NO_2_) and daily hospitalization rates for pneumonia and asthma among children aged under 5 years. Subgroup analyses were performed to evaluate associations between age, sex, season, and pathology and hospitalization rates. Hospitalization data were obtained from the big data platform of the Ningbo Health Information Center and air pollutant data were obtained from the Ningbo Air Quality Monitoring System. Our conclusions provide essential information for the study of environmental triggers of pneumonia and asthma in children in children under 5 years old and further highlight the urgency of developing effective public health interventions and help provide healthier communities.

## 2. Methods

### 2.1. Study setting

The southeast coastal city of Ningbo is situated in the southern wing of the Yangtze River Delta and possesses total land and urban areas of 9,816 and 9,816 km^2^, respectively (28°51′-30°33′N, 120°55′-122°16′E). The total sea area of Ningbo is 8,355.8 km^2^ and its total coastline length is 1,594.4 km. The Ningbo area is characterized by a subtropical monsoon climate, seasons that are clearly distinguishable, adequate sunlight, abundant rainfall and a long frost-free winter period. Chemical, textile and garment, and machinery industries constitute the majority of the well-developed secondary industry in the Ningbo area. As past studies only investigated the influences of sea transport and the chemical industry on Ningbo air pollution, we selected this city for our study.

According to an official health statistics report, in 2017 Ningbo possessed a total of 4,157 medical institutions, a total of 37,315 beds, and a total population of 8.055 million people (33, 34). As such, the number of beds per thousand persons in 2017 was 4.66. Four Class 3 Grade A general and two Class 3 Grade A specialized hospitals operated in Ningbo in 2017, exceeding standards set forth by the Outline of the National Health Service System (2015–2020). Our data were obtained from medical institutions of all levels in an effort to comprehensively evaluate hospitalization of children under 5 years of age for pneumonia or asthma.

### 2.2. Study population

Legal access to the Ningbo Health Information Center big data platform, which contained data concerning patients hospitalized for pneumonia and asthma, was provided by the Health Commission of Ningbo. Hospitalizations between January 2015 and August 2017 with principal diagnoses of pneumonia (ICD-10 codes J12–J18) and asthma (ICD-10 codes J45–J46) among children aged under 5 years were included in this study. Patients with incomplete data regarding hospital admission date or multiple versions of medical records dating from the same day were excluded from analyses. Inpatient data obtained from the big data platform included patient date of birth, sex, place of residence, disease diagnosis code, admission details and discharge date.

### 2.3. Ambient air pollution and meteorological data

Levels of ambient air pollutants recorded in urban Ningbo were obtained from the Ningbo Air Quality Data Real-time Publishing System (http://air.nbemc.net/StationAQ/Index), operated by the Ningbo Environmental Monitoring Center under direction of the Ningbo Environmental Protection Bureau and a secondary station of the National Environmental Monitoring Network. A total of 21 air quality monitoring stations throughout the metropolitan Ningbo area functioned in recording air pollution levels at the time of the study. Daily mean concentrations of PM_2.5_, PM_10_, O_3_, CO, SO_2_, and NO_2_ were averaged for each single station. For analysis of O_3_ levels, an algorithm for obtaining 8-hour moving average concentrations was used. Take 0–7 o 'clock, 1–8 o 'clock, ......, and the hourly average concentration from 17 to 24 o 'clock, respectively. The maximum value of these mean values is the mean concentration of ozone in 8 h as the daily average concentration of O_3_. For other pollutants, 24-hourly average levels were calculated. Daily average concentrations of PM_2.5_, PM_10_, SO_2_, NO_2_, and CO were calculated from the hourly concentration of a single station. Instruments at each station measure the hourly concentration of air pollutants at the station, and the 24-hour concentration value from 1 to 24 o 'clock is the arithmetic mean of the daily average concentration of air pollutants at the station. Because individual exposure levels could not be accurately assessed in this study, average levels of all pollutant measurement station data were considered as the individual exposure level as previously defined (35). Daily temperature and relative humidity data from January 1, 2015 to August 29, 2017 were obtained from the Ningbo Meteorological Bureau to control for meteorological factors (36).

### 2.4. Statistical analysis

Time series analysis is usually used to study acute health effects of air pollution; the generalized additive model (GAM) is the most widely used time series model (37). According to Hastie and Tibshirani's systematic interpretation of this model (38), GAM incorporates both parametric and non-parametric methods to identify relevant air pollutants and the presence of unknown confounding factors; known confounding factors (such as temperature and other non-linear relationships) are controlled for *via* data smoothing. Elimination of confounding factors facilitates accurate estimation of pollutant risk. Prior to model establishment, we conducted a descriptive analysis of relevant data. Spearman's correlation coefficient was used to analyze correlations between air pollutant concentrations and meteorological variables due to the non-normal distribution of data.

Because the number of daily hospitalized cases for pneumonia approximately followed a Poisson distribution, log-link function was used in model construction. Daily hospitalized pneumonia cases were considered to be a dependent variable; non-linear independent variables were time, temperature and relative humidity. As in previous studies (39–42), non-linear independent variables were modeled *via* natural cubic spline. Degrees of freedom (*df*) for each variable were selected based on partial autocorrelation function (PACF) and generalized cross-validation (GCV) values. Degrees of freedom for spline functions of non-linear independent variables were 10, 3 and 3 for time, temperature and relative humidity, respectively. Year [i.e., Year 1 (2015), Year 2 (2016), Year 3 (2017)], season (i.e., warm months, May–October; cold months, November–April) and day of week (DOW) were considered to be potential confounders in our model and treated as categorical variables. The basic form of our model was as follows:


$$\begin{array}{l} \log\left[\text{E}\left(\text{Yt}\right)\right]\text{ = }\alpha \text{+}\beta \left(\text{Pol}\right)\text{+ns}\left(\text{Time},\text{df = 10}\right)\\ \text{+ns}\left(\text{T},\text{df = 3}\right)\text{+ns}\left(\text{RH},\text{df = 3}\right)\text{+Year}\\ \text{+Season+DOW} \end{array}$$

*E(Y*_*t*_*)*, the expected number of hospitalized cases on day *t*; α, intercept; β, the log-relative risk of disease related to a unit increase in pollutant concentration; *Pol*, concentrations of pollutants; *ns( )*, natural cubic spline function; *T*, temperature; *RH*, relative humidity.

In addition to day (lag0) pollutant concentrations, we evaluated effects of pollutant concentration on lag dimension, such as effects on single-day (lag1–lag7) and multi-day (moving average, lag01–lag07) lag. Relative risks (*RR*) for 10-unit increases in PM_2.5_, PM_10_, O_3_, CO, SO_2_, and NO_2_ concentrations were reported with 95% confidence intervals (95%*CI*). Subgroup analyses were further performed to assess consequences of air pollutant levels on age (< 1 and 1–5 years), sex (boy or girl), disease (pneumonia and asthma) and season (November–April and May–October).

*RR*, excess risk (*ER*) and the 95%*CI* for hospitalizations were calculated per every 10 μg/m^3^ increase in PM_2.5_, PM_10_, SO_2_, NO_2_, and O_3_ concentrations, and per 100 μg/m^3^ increase in CO concentrations. Calculation of *ER* was performed as follows:


ER = (RR−1)×100%

Sensitivity analyses were performed accordingly: (a) to determine each pollutant's possible role, we established two-pollutant models for all pollutants without interactions; (b) cases among patients younger than 1 month old were considered to not have been influenced by exposure to pollution; and (c) to test the stability of our model, alternative *df* values (9 and 10) for time were calculated utilizing the model itself.

All statistical analyses were conducted with R version 3.6.1 for Windows. A two-tailed *p*-value <0.05 was considered statistically significant.

## 3. Results

### 3.1. Descriptive statistics of daily hospital admissions for pneumonia, pollutants and meteorological variables

In total there were 10,301 hospital admissions for pneumonia and 115 hospital admissions for asthma among children aged under 5 years in Ningbo between January 1, 2015 and August 29, 2017 (Table 1). Due to fewer cases of asthma hospitalization during the study period, we accumulated the data on a monthly basis prior to statistical description. The mean number of daily pneumonia hospitalizations was 11 (range: 1–30) and the mean number of monthly asthma hospitalizations was 4 (range: 0–13). From the quantitative perspective, the number of infant (<1 year) cases (2,634) was less than that of children aged 1–5 years (7,782) and the number of cases during colder months (November–April; 5,939 cases) was greater than that during warmer months (May–October; 4,477 cases).

**Table 1:** Descriptive statistics of the hospitalization for pneumonia and asthma among children aged < 5 years, Ningbo City, 2015–2017.

**Variable**	***N***	**Mean ±SD**	**Min**	***P_25_***	***P_50_***	***P_75_***	**Max**
**Daily hospitalizations**							
Pneumonia	10,301	10.7 ± 5.0	1	7	10	14	30
Asthma^a^	115	3.6 ± 2.8	0	1	3	5	13
**Totally daily hospitalizations by age**							
Infants (< 1 year)	2,634	2.7 ± 2.0	0	1	2	4	11
1–5 years	7,782	8.0 ± 4.0	0	5	7	11	25
**Totally daily hospitalizations by sex** ^b^							
Boy	4,730	4.9 ± 2.8	0	3	4	7	16
Girl	3,520	3.6 ± 2.3	0	2	3	5	14
**Totally daily hospitalizations by season**							
Cold (November–April)	5,939	12.3 ± 5.1	1	8	12	16	28
Warm (May–October)	4,477	9.3 ± 4.5	2	6	9	12	30

PM_2.5_, particulate matter < 2.5 μm in diameter; PM_10_, particulate matter < 10 μm in diameter.

a Asthma hospitalizations accumulate on a monthly basis;

b 2,166 cases of missing gender labels.

Statistical summaries concerning ambient air pollutant concentrations and meteorological conditions encountered throughout the study period were detailed previously (43), and included temperature and relative humidity means of 18.2°C (range: −4.5–34.8°C) and 74.8% (range: 35.0–98.0%), as well as mean PM_2.5_, PM_10_, O_3_, SO_2_, NO_2_, and CO concentrations of 40.2, 63.5, 99.3, 13.0, 39.7 μg/m^3^, and 0.8 mg/m^3^, respectively. Additionally, correlations among ambient air pollutants and their relationships with meteorological conditions were consistent with findings reported by us previously (43), with PM_2.5_, PM_10_, SO_2_, NO_2_, and CO concentrations having positively correlated with those of other pollutants (apart from O_3_) and negatively correlated with temperature.

### 3.2. Associations between air pollutants and daily hospital admissions for pneumonia and asthma

**Figure 1:**
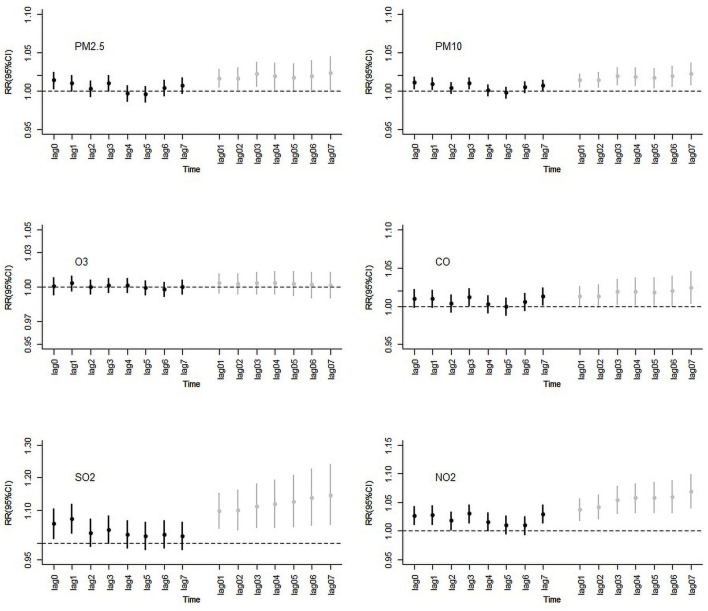
*RR* (Rate ratios) and 95%*CIs* (confidence intervals) following a 10-units increase on lag0, lags1–7, and lags01–07 ambient air pollution concentrations and hospital admissions for pneumonia and asthma, Ningbo, 2015–2017.

Figure 1 shows the associations between hospital admissions (pneumonia and asthma hospitalizations) and a 10-μg/m^3^ increase (a 100-μg/m^3^ increase for CO) on lag0 (reflecting acute effects), lags1–7 (reflecting hysteresis) and lags01–07 (reflecting cumulative hysteresis) for corresponding pollutants (Details of excess risks are provided in Supplementary Table 1). At lag0, hospital admissions were positively associated with all pollutants and statistically significant associations were observed for PM_2.5_ (*RR* = 1.014, 95%*CI*: 1.003, 1.024), PM_10_ (*RR* = 1.011, 95%*CI*: 1.003, 1.018), SO_2_ (*RR* = 1.060, 95%*CI*: 1.016, 1.106) and NO_2_ (*RR* = 1.027, 95%*CI*: 1.011, 1.043) (Supplementary Table 2). Analysis of PM_2.5_, PM_10_, SO_2_, and NO_2_ revealed effects of irregular *RR* value fluctuations for lags1–7; for lags01–07, *RR* values trended up and stabilized at lag03. In summary, the adverse effects of SO_2_ were found to be greatest among all air pollutants, with findings confirmed by both daily and the cumulative lag values.

**Figure 2:**
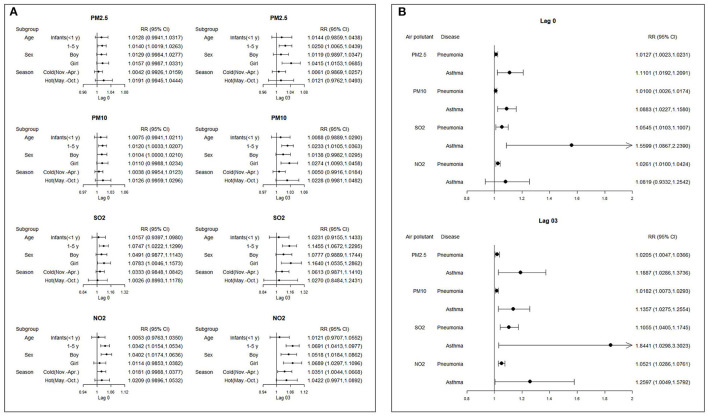
*RR* (Rate ratios) and 95% CIs (confidence intervals) following a 10-μg/m3 increase on lag0 and lag03 of all cases and different subgroups ambient air pollution concentrations and hospital admissions for pneumonia and asthma. **(A)** Subgroup analyses by age, sex and season. **(B)** Subgroup analyses by pathology).

Figure 2 presents *RR*s and 95%*CI*s according to 10-μg/m^3^ increases for lag0 and lag03 among all cases, concentrations of different air pollutant subgroups and hospitalizations. Effects of cumulative lag generally reached a stable, high level on lag03; this was true among different subgroups. For all cases, we found that hospital admissions were increased by 1.38, 1.08, 5.99, and 2.69% on lag0 per 10-μg/m^3^ increase in PM_2.5_, PM_10_, SO_2_, and NO_2_, respectively. Judging from overall lag03 values, increases were found to be 2.22, 1.94, 11.21, and 5.42%, respectively. All of these findings were statistically significant. Significant associations for asthma were found for PM_2.5_ (*RR* = 1.110, 95%*CI*: 1.019, 1.209), PM_10_ (*RR* = 1.088, 95%*CI*: 1.023, 1.158), and SO_2_ (*RR* = 1.560, 95%*CI*: 1.088, 2.239). Large *CI*s were likely noted due to the relatively small number of cases; hospital admissions for pneumonia were found to possess narrow *CIs* as well as statistical significance.

### 3.3. Subgroup analyses by age, sex, pathology and season

Significant associations for children aged 1–5 years were found for PM_2.5_ (*RR* = 1.014, 95%*CI*: 1.002, 1.026), PM_10_ (*RR* = 1.012, 95%*CI*: 1.003, 1.021), SO_2_ (*RR* = 1.075, 95%*CI*: 1.022, 1.130) and NO_2_ (*RR* = 1.034, 95%*CI*: 1.015, 1.053); no significant associations were observed for infants (aged < 1 year) for all pollutants.

The associations of ambient air pollutant concentrations and hospital admissions for pneumonia and asthma were higher during warmer as compared to colder months, apart from SO_2_, which was found to have a higher association throughout colder months. However, these differences were not significant.

### 3.4. Sensitivity analyses

**Figure 3:**
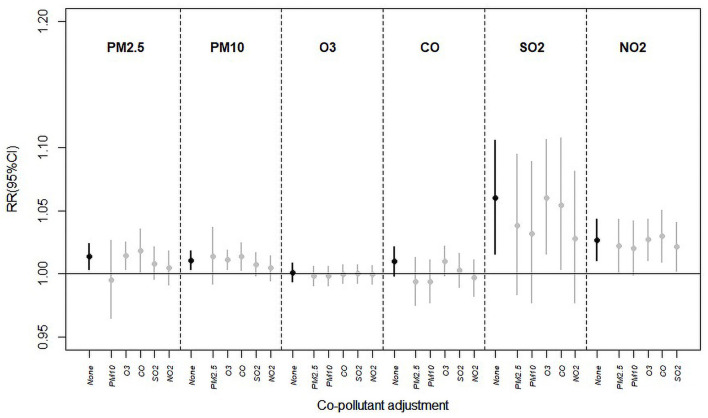
Estimated *RR* (Rate ratios) and 95%*CIs* (confidence intervals) in the single- and two-pollutant models with adjustment for PM_2.5_, PM_10_, O_3_, CO, SO_2_, and NO_2_ on lag0 for hospital admissions for pneumonia and asthma.

Estimated *RR* and 95%*CI* values in single- and two-pollutant models adjusted for PM_2.5_, PM_10_, O_3_, CO, SO_2_, and NO_2_ on lag0 for hospital admissions for pneumonia and asthma are shown in Figure 3. Addition of other pollutants to the model revealed that as compared with single-pollutant model results, estimated RRs changed to a certain extent and sometimes even lost statistical significance. Except for that of O_3_ (not statistically significant), estimated health effects of NO_2_ were found to be most stable (as determined by whether statistical significance was lost statistical) across both models. In general, estimates of PM_2.5_, PM_10_, SO_2_, and NO_2_ decreased after inclusion of other pollutants (apart from O_3_ and CO; associations between PM_10_ and hospital admissions for pneumonia and asthma increased slightly after the inclusion of PM_2.5_). Estimates of PM_2.5_, PM_10_, and SO_2_ in particular lost significance.

Supplementary Table 3 shows sensitivity analyses findings, considering PM_2.5_ as an example, by excluding children aged <1 month (total number of excluded infants: 36) and using alternative degrees of freedom (df: 9 and 10) for time. Exclusion of infants was estimated to not have significantly affected results as compared to including this population in analysis. In addition, when time df was 9 or 10, model results were generally stable.

## 4. Discussion

During the study period, mean concentrations of PM_2.5_ (40.2 ± 24.7 μg/m^3^), PM_10_ (63.5 ± 35.0 μg/m^3^), NO_2_ (39.7 ± 17.0 μg/m^3^), and O_3_ (99.3 ± 41.9 μg/m^3^) were found to have been significantly higher than standards set forth by WHO Air Quality Guidelines (44) (AQGs; PM_2.5_: 5 μg/m^3^ annual mean; PM_10_: 15 μg/m^3^ annual mean; NO_2_: 10 μg/m^3^ annual mean; O_3_:60 μg/m^3^ peak season mean) in Ningbo, China. In contrast, the concentrations of SO_2_ (13.0 ± 5.9 μg/m^3^) and CO (0.8 ± 0.2 mg/m^3^) remained within limits (SO_2_: 40 μg/m^3^ 24-h mean; CO: 4 mg/m^3^ 24-h mean). In addition, the single-day effect of the overall adverse effects of air pollutants was highest at lag0, while the cumulative lag effect was highest at lag03. To our knowledge, this is one of the few Chinese studies performed to assess effects of ambient air pollutants on the increase in the number of pneumonia and asthma hospitalizations among children aged under 5 years.

We used population-based time series analysis to confirm that increased atmospheric concentrations of PM_2.5_, PM_10_, SO_2_, and NO_2_ were significantly associated with increased hospital admissions for these illnesses among the aforementioned population. Our findings were consistent with those reported in previous studies, although specific estimates differed from study to study (45, 46). This discrepancy was likely due to differences in utilized research methods, an inherently different period considered in different studies and objective regional differences.

In the overall air pollution, the adverse effect of SO_2_ was found to be greatest among all pollutants, as confirmed by lag0 results and those of the entire cumulative lag period. The results for the same region in 2018 also showed that SO_2_ had the greatest impact on the loss of life due to ischaemic heart disease, followed by NO_2_ (47). Our results were consistent with a meta-analysis performed by Nhung et al. (48), which similarly reported SO_2_ to carry the highest excess risk among many air pollutants, with each 10 μg/m^3^ increase in SO_2_ concentration exposure associating with a 2.90% increase in pediatric pneumonia diagnoses (95%*CI*: 1.004, 1.053). Meanwhile, a previous study also performed in Ningbo similarly demonstrated that the strongest adverse effect resulting in pneumonia hospitalization was exerted by SO_2_; every 10 μg/m^3^ increase in SO_2_ exposure at lag5 was found to be associated with an increase in risk of 5.0% (95%*CI*: 1.013, 1.088) among children for pneumonia hospitalization (49). Previous studies also reported adverse effects of SO_2_ on asthma risk in children. One meta-analysis of 22 studies revealed a significant association between SO_2_ and asthma attacks in a subgroup of children (*OR* = 1.047, 95%*CI*: 1.009, 1.086) (50). In addition, a study in Taiwan reported that among all pollutants studied, SO_2_ most strongly correlated with daily asthma hospitalization in children aged 0–5 years (*RR* = 1.647, 95%*CI*: 1.607, 1.689) (51). These findings underscore the significant acute effects and delayed cumulative toxicity of SO_2_ on pediatric asthma and pneumonia hospitalization. To explore possible causes for these phenomena, several experiments investigated the mechanism by which SO_2_ exerts stronger adverse effects. A highly soluble gas that also functions as a bronchoconstrictor, SO_2_ stimulates receptor fibers and mucosal sensory nerves in the airway (52), thereby upregulating expression of proinflammatory cytokines, producing a Th1/Th2 imbalance, aggravating the pulmonary inflammatory response, promoting inflammation and increasing oxidative stress (53, 54). As such, the environmental increase in SO_2_ leads to a significant decrease in small airway function and increased airway oxidative stress, potentially manifesting with asthma (55).

Secondary to SO_2_, PM_2.5_, and PM_10_ were also found to significantly influence pediatric asthma and pneumonia hospitalizations at lag0 and lag03 in this study. The health effects of PM on the public, as well as relevant pathologic mechanisms, have been widely studied in recent decades. Particles <2.5 mm in diameter are capable of entering the circulation, while particles <10 mm in diameter are capable of penetrating deep into pulmonary tissue. Various PM components such as organic carbon, nitrates, metals and silicon have been linked to development of early-life pneumonia (56). Fine PM likely exerts stronger effects on lung cells due to deeper tissue deposition and slower clearance (57). Importantly, PMs produce free radicals and cause oxidative stress in lung cells (15). In addition, PMs increase susceptibility to infection *via* suppression of immunity (12). Effects of PM on pneumonia have also been widely studied (48, 58, 59). One systematic meta-analysis concluded that for each 10 mg/m^3^ increase in PM_2.5_ and PM_10_, short-term impacts on hospitalization for pediatric pneumonia in children were ~1.8% (95%*CI*: 1.005, 1.031) and 1.5% (95%*CI*: 1.006, 1.024), with PM_2.5_ second only to SO_2_ in terms of influence on risk (48). A study performed in Qingdao, China reported that an increase in interquartile spacing between PM_2.5_ and PM_10_ was associated with a significant increase in risk of hospitalization for pneumonia in children under 4 years of age, at 7.5% (95%*CI*: 1.017, 1.136) and 10.1% (95%*CI*: 1.037, 1.169), respectively (58). Taiwanese studies have provided higher estimates, reporting increased interquartile spacing of PM_2.5_ and PM_10_ at lag3 to increase the risk for emergency department admission for children suffering pneumonia by 18.2 (95%*CI*: 1.088, 1.284) and 13.1% (95%*CI*: 1.051, 1.217) (59). Moreover, the adverse effects of PM on pediatric hospitalization for respiratory diseases such as asthma and bronchiolitis were also studied in Brazil (60) and the United States (61). The two-pollutant model utilized in this study revealed that PM_2.5_ and PM_10_ lost some statistical significance after introduction of NO_2_ and SO_2_ to analyses; it can thus be speculated that pediatric pneumonia and asthma hospitalization are influenced more greatly by NO_2_ and SO_2_ concentrations.

Surprisingly, we found that NO_2_ exerted the most consistent effect on hospitalization according to both of our pollutant models. However, adverse effects of NO_2_ were noted to decrease after introduction of SO_2_ and PM to analysis, with findings retaining statistical significance. Similar findings were reported in an Australian study where the effect of outdoor air NO_2_ content on pediatric hospitalizations was found to be largely independent of effects exerted by other pollutants (62). One Vietnamese study reported that an increase in NO_2_ levels in the mean quartile range (21.9 μg/m^3^) was associated with a 6.1% increase in pediatric hospitalizations for pneumonia (95%*CI*: 1.025, 1.098), with findings remaining consistent in analyses of multiple pollutants (21). Those findings are likely explained by NO_2_ being a free radical whose action has the potential to deplete tissue antioxidant defenses and lead to increased inflammation. Furthermore, exposure to NO_2_ was associated with reduced lung volume growth (63) an increased pneumonia incidence (64–66) and increased penicillin MIC against Streptococcus pneumoniae among children (67). In addition, prenatal and postnatal exposure to NO_2_ was found to be a likely risk factor for pediatric allergies and respiratory diseases (68). Therefore, compared with other pollutants, NO_2_ seems to better reflect the effects of air pollution on pediatric pneumonia and asthma hospitalization. Considering the stability of NO_2_, and its properties as a major traffic pollutant (69, 70), implementation of more effective environmental policies should be formulated in Ningbo to reduce children's exposure to NO_2_ and thus its harmful effects on childhood health.

Age specific analysis revealed the adverse effects of air pollutants on pediatric pneumonia and asthma to be higher as compared to infants. Similar results have been reported in other Chinese cities (71–73) as well as some foreign cities (74, 75). These results may be related to the behavior patterns of children and the effects of breast milk. Importantly, infants under the age of 1 year usually have full-time caregivers that nurture them exclusively indoors, while older children (2–5 years) are generally more active and thus more exposed to outdoor air pollutants (75). Interestingly, breastfeeding was reported to be an important protective factor for a child's immune system against air pollution. Several studies in China have shown that breastfeeding is associated with a reduced risk of pediatric respiratory diseases (76–78). Hence, Infants received more protection from breast milk than children 1–5 years of age. These findings suggest that parents should pay attention to the respiratory health of children aged 1–5 years who are vulnerable to air pollution, and take measures such as reducing travel or wearing masks on smoggy days to reduce the harm of air pollution to children. In addition, the care and protection of infants under 1 year old should be maintained, and breastfeeding should be advocated.

Our study was not without some seasonal heterogeneity. Generally, the adverse effects of PM_2.5_, PM_10_, and NO_2_ were noted to be slightly higher in warmer as compared to colder months, consistent with findings reported by previous studies performed in Beijing (79), Fuzhou (80), Taiwan (81), and Hong Kong (82). The reasons why air pollutants are more harmful in a warmer season can be explained by high temperature itself being associated with increased risk of pneumonia (83). Furthermore, higher temperatures affect emission, transportation, dilution, chemical conversion, and deposition of air pollutants (84). In addition, significant thermoregulatory stress to heat, manifesting in processes such as sweating, similarly increases absorption and distribution of air pollutants in the human body (85). Effects observed may also be explained by temperature-influenced patterns of exposure; namely people tending to engage in greater frequencies of outdoor activities in warmer months and thus increasing air pollutant exposure (86). However, the influences of temperature remain inconclusive, with some studies having reported air pollutants to more markedly increase pneumonia hospitalization rates in colder months (87). Further research is thus warranted needed to investigate the impact of temperature on air pollutants and health.

Sex subgroup analysis revealed that apart from NO_2_, the adverse effects of PM_2.5_, PM_10_, and SO_2_ on pneumonia and asthma were higher in girls as compared to boys. One similar subgroup analysis from Wuhan reported that PM_2.5_ had a higher *OR* of 1.020 (95%*CI*: 1.007, 1.034) in hospitalized females as compared to hospitalized males, while NO_2_ exerted significant effects exclusively on boys (88). Our findings similarly underscored a greater adverse effect of NO_2_ on pneumonia and asthma hospitalization rates among boys at lag0. In a Hong Kong study, PM was reported to exert a slightly greater effect on increased hospitalization rates for pneumonia among girls as compared to boys (27). One Chinese study analyzing deaths among children under 5 years of age also reported a significant correlation between exposure to PM_2.5_ and pneumonia mortality, with a larger effect estimated among girls. One birth cohort study performed in the Netherlands reported that asthma rates in girls tended to more strongly correlate with air pollution levels as compared to those of boys, although interactions between exposure and sex were not statistically significant (89). Conversely, some studies have reported boys to be more sensitive to air pollution (71, 73, 90). Although sex differences in air pollution epidemiology have recently become a hot topic of discussion, no consensus has been reached to date (90). Although exact causative mechanisms remain unclear, studies have reported boys and girls to respond differently to various air pollutants, likely due to different degrees of sensitivity to air pollution and differences in early postnatal respiratory development (90, 91). Importantly, differences in lung structure, immune processes and inflammatory responses were reported to make girls more susceptible to infection (92, 93). In addition, as boys are generally more active more frequently exposed to outdoor air pollutants than girls, this also likely results in greater risk of pulmonary illness (94). Whether there is a difference between boys and girls aged under 5 years old in outdoor activity participation in Ningbo warrants exploration.

Importantly, we found that the air pollutants analyzed in this study exerted much greater adverse effects on asthma as compared with pneumonia hospitalization. Although previous studies conducted in Sri Lanka (95), Georgia (96), and the United States (97) reported similar findings and underscored a positive correlation between air pollutants such as PM and NO_2_ and asthma hospitalization in the case of asthma, either no significant correlation with pneumonia hospitalization or loss of significance after model adjustment found in the case of pneumonia. In Vietnam, study of high air pollutant concentrations revealed that although PM_2.5_, PM_10_, SO_2_, and NO_2_ were positively correlated with hospitalization for pneumonia and asthma, estimated risk of air pollutants for asthma became greater while estimated risk for pneumonia decreased after adjustment for PM_2.5_ (21). Oxidative stress, airway remodeling, inflammation, immune dysregulation and enhanced respiratory sensitization to airborne allergens have all been proposed to explain how air pollution contributes to asthma pathogenesis (98). The occurrence and exacerbation of asthma are also influenced by various factors such as genetic susceptibility and ancestry (99), recent viral respiratory tract infection or allergen exposure (100), extreme weather (101) and pollen exposure (102, 103), among others. Interestingly, prior findings suggest an increased susceptibility to asthma as compared to pneumonia. Furthermore, a study evaluating children in 18 European countries reported 33% more cases of childhood asthma to be attributable to air pollution exposure (PM_2.5_) (104). In light of the relatively small number of asthma cases in our study, future correlation analyses are warranted. In addition, adoption of more appropriate policies to reasonably reduce exposure to air pollutants and thus asthma incidence among children are necessary.

Our study was not without limitations. Firstly, like other research in the field of environmental health relying on use of large health databases, data on patient-level variables were not available, which limited our ability to identify potentially more susceptible populations. More detailed individual data would be required to correct for confounding effects of individual characteristics on the associations between air pollutants and childhood pneumonia and asthma hospitalization. As our sample size of asthma hospital admission was limited, the validity of our conclusions concerning relationships between asthma and air pollutants remains in question. Finally, as emergency room visits for pneumonia and asthma were not analyzed in this study, future research is warranted to more accurately assess the impact of air pollutants on emergency department admission in the context of acute pulmonary conditions such as pneumonia and asthma.

Of note, this study possessed several points of strength. Firstly, our findings revealed detailed estimates of the risk of air pollutant-associated hospital admission in the settings of pediatric pneumonia and asthma utilizing high-quality data obtained from a big data platform that included clinical information of all children residing in Ningbo. Secondly, successful construction of our analytical model allowed for performance of reasonable subgroup and sensitivity analyses elucidating the detailed impact of air pollutants on pediatric pulmonary health. Finally, our utilization of high-quality meteorological data allowed for representative estimation of effects expected to be exerted by air pollutants on health throughout the Ningbo metropolitan area.

## 5. Conclusion

Exposure of air pollutants including PM_2.5_, PM_10_, SO_2_, and NO_2_ in the Ningbo metropolitan area was found to positively correlate with pneumonia- and asthma-related hospitalizations among children aged under 5 years. Among these pollutants, NO_2_ likely remains the most stable and exerts the most significant adverse effects on human health. Adverse effects were found to be stronger in children aged 1–5, with adverse effects of PM_2.5_, PM_10_, and SO_2_ generally stronger in girls. Implementation of more appropriate public health measures throughout the urban Ningbo area is warranted to reduce childhood exposure to PM_2.5_, PM_10_, SO_2_, and NO_2_, and in turn reduce rates of pediatric hospitalizations for acute pulmonary conditions.

## Data availability statement

The original contributions presented in the study are included in the article/Supplementary material, further inquiries can be directed to the corresponding authors.

## Author contributions

Conceptualization: XZho and LL. Methodology: XZha and MG. Software: GH. Formal analysis: MG. Data curation: ZhiL and XY. Writing-original draft preparation: LL and XZho. Writing-review and editing: XZho, MG, ZheL, and XZha. Funding acquisition: LL. All authors contributed to the article and approved the submitted version.

## Supplementary material

Supplementary Table 1 *ER* (Excess risk) and 95%*CIs* (confidence intervals) following a 10-units increase on lag0, lags1–7 and lags01–07 ambient air pollution concentrations and hospital admissions for pneumonia and asthma, Ningbo, 2015–2017.

Supplementary Table 2 Association between different air pollutants and increases of 10-μg/m^3^ (CO increase of 100-μg/m^3^) and hospitalization rates (hospitalization for pneumonia and asthma).

Supplementary Table 3 A sensitivity analysis: excluding children under 1 month of age and different degrees of freedom of time variables (taking PM_2.5_ as an example).
