# Supplementary material for: Associations between air pollutant and pneumonia and asthma requiring hospitalization among children aged under 5 years in Ningbo, 2015–2017
Source: Front Public Health. 2023 Jan 25;10:1017105. doi: 10.3389/fpubh.2022.1017105 (PMC9908005; doi:10.3389/fpubh.2022.1017105)
Supplement: Supplementary Table 1 — ER (Excess risk) and 95%CIs (confidence intervals) following a 10-units increase on lag0, lags1–7 and lags01–07 ambient air pollution concentrations and hospital admissions for pneumonia and asthma, Ningbo, 2015–2017. [file Data_Sheet_1.zip › Supplementary Table 1.docx]

**Supplementary Table 1.** *ER* (Excess risk) and 95%*CIs* (confidence intervals) following a 10-units increase on lag0, lags1-7 and lags01-07 ambient air pollution concentrations and hospital admissions for pneumonia and asthma, Ningbo, 2015–2017.

| **Lag** | **PM_2.5_** | | | **PM_10_** | | | **O_3_** | | | **CO** | | | **SO_2_** | | | **NO_2_** | | |
| --- | --- | --- | --- | --- | --- | --- | --- | --- | --- | --- | --- | --- | --- | --- | --- | --- | --- | --- |
|  | ***ER*** | **95%*CI*** | | ***ER*** | **95%*CI*** | | ***ER*** | **95%*CI*** | | ***ER*** | **95%*CI*** | | ***ER*** | **95%*CI*** | | ***ER*** | **95%*CI*** | |
| Lag0 | 1.38 | 0.35 | 2.41 | 1.08 | 0.35 | 1.82 | 0.10 | -0.65 | 0.84 | 0.99 | -0.16 | 2.17 | 5.99 | 1.57 | 10.61 | 2.69 | 1.08 | 4.31 |
| Lag1 | 1.03 | 0.02 | 2.06 | 0.96 | 0.24 | 1.69 | 0.33 | -0.32 | 0.99 | 1.01 | -0.12 | 2.15 | 7.52 | 3.22 | 12.00 | 2.72 | 1.09 | 4.38 |
| Lag2 | 0.26 | -0.77 | 1.29 | 0.40 | -0.33 | 1.13 | 0.03 | -0.59 | 0.65 | 0.39 | -0.73 | 1.52 | 3.12 | -1.03 | 7.45 | 1.79 | 0.22 | 3.39 |
| Lag3 | 0.98 | -0.04 | 2.02 | 0.98 | 0.26 | 1.70 | 0.14 | -0.48 | 0.76 | 1.20 | 0.07 | 2.34 | 4.09 | -0.08 | 8.44 | 3.02 | 1.47 | 4.59 |
| Lag4 | -0.32 | -1.33 | 0.72 | 0.08 | -0.64 | 0.80 | 0.15 | -0.47 | 0.77 | 0.28 | -0.86 | 1.43 | 2.72 | -1.42 | 7.04 | 1.62 | 0.08 | 3.19 |
| Lag5 | -0.39 | -1.41 | 0.64 | -0.17 | -0.89 | 0.55 | -0.09 | -0.71 | 0.53 | -0.03 | -1.17 | 1.12 | 2.21 | -1.90 | 6.49 | 1.07 | -0.47 | 2.64 |
| Lag6 | 0.38 | -0.65 | 1.41 | 0.47 | -0.25 | 1.19 | -0.21 | -0.82 | 0.41 | 0.53 | -0.60 | 1.68 | 2.74 | -1.39 | 7.03 | 0.96 | -0.57 | 2.52 |
| Lag7 | 0.71 | -0.30 | 1.74 | 0.66 | -0.06 | 1.38 | 0.00 | -0.61 | 0.62 | 1.31 | 0.18 | 2.45 | 2.15 | -1.98 | 6.45 | 2.99 | 1.44 | 4.56 |
|  |  |  |  |  |  |  |  |  |  |  |  |  |  |  |  |  |  |  |
| Lag01 | 1.67 | 0.46 | 2.90 | 1.40 | 0.54 | 2.27 | 0.34 | -0.51 | 1.19 | 1.31 | 0.00 | 2.65 | 9.76 | 4.47 | 15.31 | 3.69 | 1.79 | 5.62 |
| Lag02 | 1.63 | 0.23 | 3.06 | 1.46 | 0.47 | 2.45 | 0.27 | -0.63 | 1.19 | 1.33 | -0.16 | 2.83 | 10.03 | 4.14 | 16.27 | 4.19 | 2.05 | 6.39 |
| Lag03 | 2.22 | 0.64 | 3.81 | 1.94 | 0.85 | 3.05 | 0.33 | -0.63 | 1.30 | 1.90 | 0.26 | 3.56 | 11.21 | 4.70 | 18.12 | 5.42 | 3.07 | 7.82 |
| Lag04 | 1.96 | 0.24 | 3.70 | 1.87 | 0.69 | 3.07 | 0.38 | -0.63 | 1.40 | 1.91 | 0.15 | 3.71 | 11.88 | 4.80 | 19.43 | 5.74 | 3.23 | 8.31 |
| Lag05 | 1.70 | -0.16 | 3.59 | 1.70 | 0.44 | 2.99 | 0.30 | -0.75 | 1.37 | 1.80 | -0.08 | 3.72 | 12.66 | 4.99 | 20.89 | 5.86 | 3.20 | 8.59 |
| Lag06 | 1.93 | -0.07 | 3.96 | 1.91 | 0.56 | 3.27 | 0.18 | -0.92 | 1.30 | 1.97 | -0.02 | 3.99 | 13.78 | 5.52 | 22.69 | 5.95 | 3.15 | 8.82 |
| Lag07 | 2.35 | 0.22 | 4.52 | 2.20 | 0.77 | 3.65 | 0.17 | -0.97 | 1.32 | 2.45 | 0.37 | 4.57 | 14.58 | 5.77 | 24.13 | 6.89 | 3.96 | 9.90 |

Abbreviations: Calculation of *ER* (Excess risk) was performed as follows: ER=(RR-1) ×100%
