# Supplementary material for: Associations between air pollutant and pneumonia and asthma requiring hospitalization among children aged under 5 years in Ningbo, 2015–2017
Source: Front Public Health. 2023 Jan 25;10:1017105. doi: 10.3389/fpubh.2022.1017105 (PMC9908005; doi:10.3389/fpubh.2022.1017105)
Supplement: Supplementary Table 1 — ER (Excess risk) and 95%CIs (confidence intervals) following a 10-units increase on lag0, lags1–7 and lags01–07 ambient air pollution concentrations and hospital admissions for pneumonia and asthma, Ningbo, 2015–2017. [file Data_Sheet_1.zip › Supplementary Table 2.docx]

**Supplementary Table 2.** Association between different air pollutants and increases of 10-μg/m^3^ (CO increase of 100-μg/m^3^) and hospitalization rates (hospitalization for pneumonia and asthma)

| **Lag** | **PM_2.5_** | | | **PM_10_** | | | **O_3_** | | | **CO** | | | **SO_2_** | | | **NO_2_** | | |
| --- | --- | --- | --- | --- | --- | --- | --- | --- | --- | --- | --- | --- | --- | --- | --- | --- | --- | --- |
|  | ***RR*** | **95%*CI*** | | ***RR*** | **95%*CI*** | | ***RR*** | **95%*CI*** | | ***RR*** | **95%*CI*** | | ***RR*** | **95%*CI*** | | ***RR*** | **95%*CI*** | |
| Lag0 | **1.014** | **1.003** | **1.024** | **1.011** | **1.003** | **1.018** | 1.001 | 0.994 | 1.008 | 1.010 | 0.998 | 1.022 | **1.060** | **1.016** | **1.106** | **1.027** | **1.011** | **1.043** |
| Lag1 | **1.010** | **1.000** | **1.021** | **1.010** | **1.002** | **1.017** | 1.003 | 0.997 | 1.010 | 1.010 | 0.999 | 1.022 | **1.075** | **1.032** | **1.120** | **1.027** | **1.011** | **1.044** |
| Lag2 | 1.003 | 0.992 | 1.013 | 1.004 | 0.997 | 1.011 | 1.000 | 0.994 | 1.007 | 1.004 | 0.993 | 1.015 | 1.031 | 0.990 | 1.075 | **1.018** | **1.002** | **1.034** |
| Lag3 | 1.010 | 0.999 | 1.020 | 1.010 | 1.003 | 1.017 | 1.001 | 0.995 | 1.008 | **1.012** | **1.001** | **1.023** | 1.040 | 0.999 | 1.084 | **1.030** | **1.015** | **1.046** |
| Lag4 | 0.997 | 0.987 | 1.007 | 1.001 | 0.994 | 1.008 | 1.001 | 0.995 | 1.008 | 1.003 | 0.991 | 1.014 | 1.027 | 0.986 | 1.070 | **1.016** | **1.001** | **1.032** |
| Lag5 | 0.996 | 0.986 | 1.006 | 0.998 | 0.991 | 1.006 | 0.999 | 0.993 | 1.005 | 1.000 | 0.988 | 1.011 | 1.022 | 0.981 | 1.065 | 1.011 | 0.995 | 1.026 |
| Lag6 | 1.004 | 0.994 | 1.014 | 1.005 | 0.997 | 1.012 | 0.998 | 0.992 | 1.004 | 1.005 | 0.994 | 1.017 | 1.027 | 0.986 | 1.070 | 1.010 | 0.994 | 1.025 |
| Lag7 | 1.007 | 0.997 | 1.017 | 1.007 | 0.999 | 1.014 | 1.000 | 0.994 | 1.006 | **1.013** | **1.002** | **1.025** | 1.021 | 0.980 | 1.064 | **1.030** | **1.014** | **1.046** |
|  |  |  |  |  |  |  |  |  |  |  |  |  |  |  |  |  |  |  |
| Lag01 | **1.017** | **1.005** | **1.029** | **1.014** | **1.005** | **1.023** | 1.003 | 0.995 | 1.012 | 1.013 | 0.999 | 1.027 | **1.098** | **1.045** | **1.153** | **1.037** | **1.018** | **1.056** |
| Lag02 | **1.016** | **1.002** | **1.031** | **1.015** | **1.005** | **1.025** | 1.003 | 0.994 | 1.012 | 1.013 | 0.998 | 1.028 | **1.100** | **1.041** | **1.163** | **1.042** | **1.020** | **1.064** |
| Lag03 | **1.022** | **1.006** | **1.038** | **1.019** | **1.009** | **1.030** | 1.003 | 0.994 | 1.013 | **1.019** | **1.003** | **1.036** | **1.112** | **1.047** | **1.181** | **1.054** | **1.031** | **1.078** |
| Lag04 | **1.020** | **1.002** | **1.037** | **1.018** | **1.007** | **1.031** | 1.004 | 0.994 | 1.014 | **1.019** | **1.001** | **1.037** | **1.119** | **1.048** | **1.194** | **1.057** | **1.032** | **1.083** |
| Lag05 | 1.017 | 0.999 | 1.036 | **1.017** | **1.004** | **1.030** | 1.003 | 0.992 | 1.014 | 1.018 | 0.999 | 1.037 | **1.127** | **1.050** | **1.209** | **1.059** | **1.032** | **1.086** |
| Lag06 | 1.019 | 0.999 | 1.040 | **1.019** | **1.006** | **1.033** | 1.002 | 0.991 | 1.013 | 1.020 | 0.999 | 1.040 | **1.138** | **1.055** | **1.227** | **1.059** | **1.032** | **1.088** |
| Lag07 | **1.023** | **1.002** | **1.045** | **1.022** | **1.008** | **1.037** | 1.002 | 0.990 | 1.013 | **1.024** | **1.004** | **1.046** | **1.146** | **1.058** | **1.241** | **1.069** | **1.040** | **1.099** |

Abbreviations: df _TEM_ = 3; df _RH_ = 3; df _time_ =10; TEM: daily average temperature; RH: relative humidity.
