# Supplementary material for: Associations between air pollutant and pneumonia and asthma requiring hospitalization among children aged under 5 years in Ningbo, 2015–2017
Source: Front Public Health. 2023 Jan 25;10:1017105. doi: 10.3389/fpubh.2022.1017105 (PMC9908005; doi:10.3389/fpubh.2022.1017105)
Supplement: Supplementary Table 1 — ER (Excess risk) and 95%CIs (confidence intervals) following a 10-units increase on lag0, lags1–7 and lags01–07 ambient air pollution concentrations and hospital admissions for pneumonia and asthma, Ningbo, 2015–2017. [file Data_Sheet_1.zip › Supplementary Table 3.docx]

**Supplementary Table 3.** A sensitivity analysis: Excluding children under 1 month of age and different degrees of freedom of time variables (taking PM_2.5_ as an example)

| PM_2.5_ | Lag | *RR* (95% *CI*) | |
| --- | --- | --- | --- |
|  |  | ***df* _TEM_ = 3**  ***df* _RH_ = 3**  ***df* _time_ = 9** | ***df* _TEM_ = 3**  ***df* _RH_ = 3**  ***df* _time_ =10** |
| including children <1 month old | Lag 0 | **1.012(1.001,1.022)** | **1.014(1.003,1.024)** |
|  | Lag 1 | 1.008(0.998,1.018) | 1.010(1.000,1.021) |
|  | Lag 2 | 1.000(0.990,1.010) | 1.003(0.992,1.013) |
|  | Lag 3 | 1.007(0.997,1.017) | 1.010(0.999,1.020) |
|  | Lag 4 | 0.994(0.984,1.004) | 0.997(0.987,1.007) |
|  | Lag 5 | 0.993(0.983,1.004) | 0.996(0.986,1.006) |
|  | Lag 6 | 1.001(0.991,1.011) | 1.004(0.994,1.014) |
|  | Lag 7 | 1.004(0.994,1.014) | 1.007(0.997,1.017) |
|  | Lag 01 | **1.013(1.001,1.026)** | **1.017(1.005,1.029)** |
|  | Lag 02 | 1.012(0.998,1.026) | **1.016(1.002,1.031)** |
|  | Lag 03 | **1.016(1.000,1.031)** | **1.022(1.006,1.038)** |
|  | Lag 04 | 1.012(0.995,1.029) | **1.020(1.002,1.037)** |
|  | Lag 05 | 1.008(0.990,1.026) | 1.017(0.998,1.036) |
|  | Lag 06 | 1.008(0.989,1.028) | 1.019(0.999,1.040) |
|  | Lag 07 | 1.010(0.990,1.031) | **1.023(1.002,1.045)** |
| excluding children <1 month old | Lag 0 | **1.012(1.002,1.022)** | **1.014(1.004,1.024)** |
|  | Lag 1 | 1.008(0.998,1.018) | **1.011(1.000,1.021)** |
|  | Lag 2 | 1.000(0.990,1.010) | 1.003(0.992,1.013) |
|  | Lag 3 | 1.007(0.997,1.017) | 1.010(0.999,1.020) |
|  | Lag 4 | 0.995(0.984,1.005) | 0.997(0.987,1.008) |
|  | Lag 5 | 0.993(0.983,1.003) | 0.996(0.986,1.006) |
|  | Lag 6 | 1.000(0.990,1.011) | 1.003(0.993,1.014) |
|  | Lag 7 | 1.004(0.994,1.014) | 1.007(0.997,1.017) |
|  | Lag 01 | **1.014(1.002,1.026)** | **1.017(1.005,1.029)** |
|  | Lag 02 | 1.012(0.998,1.026) | **1.017(1.003,1.031)** |
|  | Lag 03 | **1.016(1.001,1.032)** | **1.023(1.007,1.039)** |
|  | Lag 04 | 1.012(0.995,1.029) | **1.020(1.003,1.038)** |
|  | Lag 05 | 1.008(0.990,1.026) | 1.018(0.999,1.037) |
|  | Lag 06 | 1.008(0.989,1.028) | 1.020(0.999,1.040) |
|  | Lag 07 | 1.010(0.990,1.031) | **1.024(1.002,1.045)** |

Abbreviations: TEM: daily average temperature; RH: relative humidity.
